# Roles of Lysyl Oxidase Family Members in the Tumor Microenvironment and Progression of Liver Cancer

**DOI:** 10.3390/ijms21249751

**Published:** 2020-12-21

**Authors:** Hung-Yu Lin, Chia-Jung Li, Ya-Ling Yang, Ying-Hsien Huang, Ya-Tze Hsiau, Pei-Yi Chu

**Affiliations:** 1 Research Assistant Center, Show Chwan Memorial Hospital, Changhua 500, Taiwan; linhungyu700218@gmail.com; 2 Department of Obstetrics and Gynecology, Kaohsiung Veterans General Hospital, Kaohsiung 813, Taiwan; nigel6761@gmail.com; 3 Institute of BioPharmaceutical Sciences, National Sun Yat-sen University, Kaohsiung 804, Taiwan; 4 Department of Anesthesiology, Kaohsiung Chang Gung Memorial Hospital and Chang Gung University College of Medicine, Kaohsiung 833, Taiwan; inr453@cgmh.org.tw; 5 Department of Pediatrics, Kaohsiung Chang Gung Memorial Hospital and Chang Gung University College of Medicine, Kaohsiung 833, Taiwan; yhhuang123@yahoo.com.tw; 6 Department of Pathology, Show Chwan Memorial Hospital, Changhua 500, Taiwan; 7 School of Medicine, College of Medicine, Fu Jen Catholic University, New Taipei City 242, Taiwan; 8 Department of Health Food, Chung Chou University of Science and Technology, Changhua 510, Taiwan; 9 National Institute of Cancer Research, National Health Research Institutes, Tainan 704, Taiwan

**Keywords:** liver cancer, hepatocellular carcinoma, cholangiocarcinoma, lysyl oxidase family members, tumor microenvironment

## Abstract

The lysyl oxidase (LOX) family members are secreted copper-dependent amine oxidases, comprised of five paralogues: LOX and LOX-like l-4 (LOXL1-4), which are characterized by catalytic activity contributing to the remodeling of the cross-linking of the structural extracellular matrix (ECM). ECM remodeling plays a key role in the angiogenesis surrounding tumors, whereby a corrupt tumor microenvironment (TME) takes shape. Primary liver cancer includes hepatocellular carcinoma (HCC) and cholangiocarcinoma (CCA), ranked as the seventh most common cancer globally, with limited therapeutic options for advanced stages. In recent years, a growing body of evidence has revealed the key roles of LOX family members in the pathogenesis of liver cancer and the shaping of TME, indicating their notable potential as therapeutic targets. We herein review the clinical value and novel biological roles of LOX family members in tumor progression and the TME of liver cancers. In addition, we highlight recent insights into their mechanisms and their potential involvement in the development of target therapy for liver cancer.

## 1. Introduction

**Figure 1:**
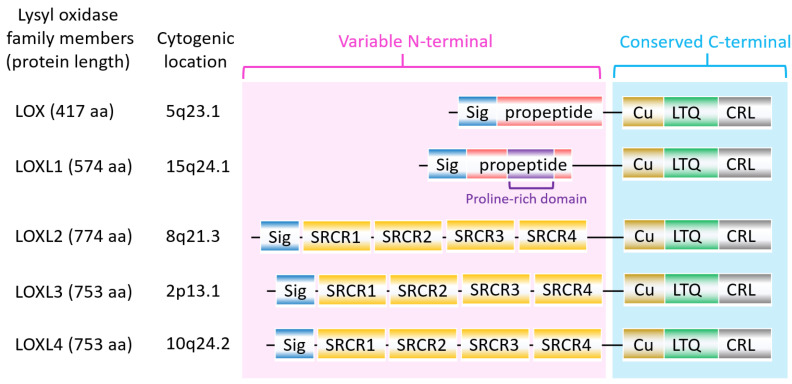
The structure of lysyl oxidase (LOX) family members. LOX family members encoded by the human LOX/LOXLs genes are located at various chromosome sites, including 5q23.1, 15q24.1, 8p21.3, 2p13.1, and 10q24.2. These members consist of a variable N-terminal domain and a highly conserved C-terminal domain. Sig, signal peptide (Sig); copper binding domain (Cu); lysyl-tyrosyl-quinone (LTQ) co-factor; scavenger receptor cysteine-rich (SRCR) domain; cytokine receptor-like (CRL) domain.

**Figure 2:**
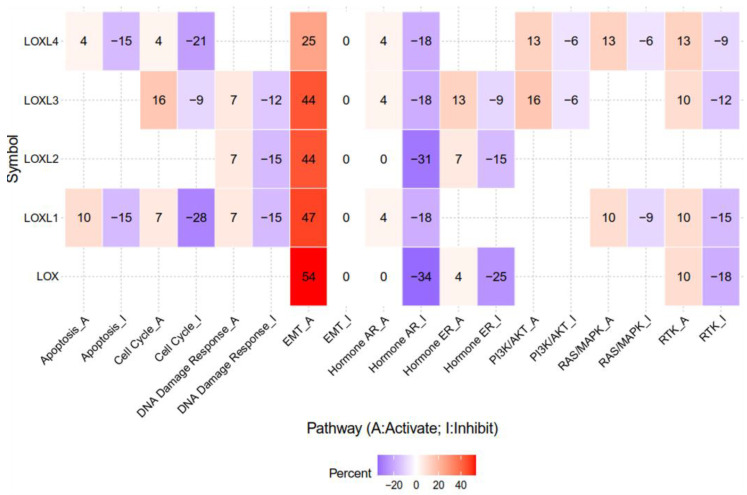
The epithelial–mesenchymal transition (EMT) pathway is activated by LOX, LOXL1, LOXL2, LOXL3, and LOXL4 in genomic big data-centric pathway analysis. Heatmap data demonstrated that LOX, LOXL1, LOXL2, LOXL3, and LOXL4 have activating/inhibiting (red/blue) functions on each cancer-related pathway. Note that LOX, LOXL1, LOXL2, LOXL3, and LOXL4 account for 54%, 47%, 44%, 44%, and 25% of cancers in the EMT-activating pathway, respectively. The pathway activity module was assessed with the GSCALite web server. High-throughput antibody-based technique reverse phase protein array (RPPA) was conduct to determine the expression of The Cancer Genome Atlas (TCGA) samples of at least 5 cancer types. Known cancer-related pathways are included: TSC/mTOR, RTK, RAS/MAPK, PI3K/AKT, hormone ER, hormone AR, EMT, DNA damage response, cell cycle, apoptosis.

The lysyl oxidase (LOX) family members are secreted copper-dependent amine oxidases, comprised of five paralogues: LOX and LOX-like l-4 (LOXL1-4) [1]. As shown in Figure 1, LOX family members encoded by the human LOX/LOXLs genes are located at various chromosome sites, including 5q23.1, 15q24.1, 8p21.3, 2p13.1, and 10q24.2 [2,3]. These members structurally consist of a variable N-terminal domain and a highly conserved C-terminal domain (Figure 1). The conserved C- terminal consists of copper binding domain amino acid residues forming lysine tryosylquinone (LTQ), and a cytokine receptor-like (CRL) domain [4]. In the N-terminal domain, LOX and LOXL1 possess a propeptide sequence, whereas LOXL2–4 present four scavenger-receptor cysteine-rich (SRCR) domains in this region [5]. The matured active forms of LOX and LOXL1 are formed by a cleavage process executed by bone morphogenetic protein 1 (BMP-1), which is not a required program for LOXL2, LOXL3, or LOXL4 [6] (Figure 1). LOX family members are characterized by their catalytic activity contributing to structural integrity and increased tensile strength, acting to remodel the cross-linking of the structural extracellular matrix (ECM) of fibrotic organs such as the liver [7,8,9,10], as well as that of the cancer microenvironments [2,4]. A growing body of evidence indicates that the expression of LOX family members increases in invasive and metastatic cancers, and their elevated expression correlates with poor survival [11,12,13]. Their crucial role in tumor proliferation, epithelial–mesenchymal transition (EMT), migration, invasion, formation of pre-metastatic niches, and immunomodulation have been well documented [11,14,15,16,17]. Consistent with these reports, we note that a genomic big data-centric pathway activity analysis reveals their role in the activation of the EMT pathway in cancer (Figure 2).

Primary liver cancer is ranked as the seventh most common cancer globally, including hepatocellular carcinoma (HCC) and cholangiocarcinoma (CCA) [18]. HCC accounts for approximately 90%, while CCA and the combination of HCC and CCA account for 10% of liver cancers [19]. HCC features hepatocellular characteristics at the morphological and molecular levels, whereas CCA exhibits biliary epithelial cell properties. Of note, limited therapeutic options are currently available for the advanced stages of liver cancers. Furthermore, as estimated by the World Health Organization (WHO), more than one million patients are projected to die from liver cancer in the next decade [20]. Growing evidence supports the role of the tumor microenvironment (TME) in the development and progression of HCC. The TME is composed of cellular and non-cellular components. Cellular components include angiogenic endothelial cells, immune system cells, tumor-associated fibroblasts (TAF), and tumor-associated macrophage (TAM); while non-cellular components involve ECM, exosomes, soluble cytokines, and signaling molecules [21].

Mounting evidence in recent years has revealed the key roles of LOX family members in the pathogenesis of liver cancer. Their ECM-remodeling and secretable nature permits the shaping of TME in both the primary organ and distal metastatic sites. More importantly, their potential as therapeutic targets is notably emerging. This expeditious progress prompted us to summarize the prognostic significance of such research (Table 1), and to review the novel biological roles of LOX family members in tumor cells and the TME of liver cancer. Furthermore, we highlight recent insights into their mechanisms and potential for target therapy approaches.

## 2. Role of LOX in Liver Cancer

### 2.1. Prognostic Value and Biological Role of LOX in HCC

LOX mRNA encodes pre-pro-LOX protein which is subsequently transformed into inactive pro-LOX protein in the cytoplasm. The pro-LOX protein is then cleaved by BMP-1, resulting in an active LOX protein and LOX peptide (LOX-PP). Elevated expressions of the LOX level have been noted in HCC tissue compared to that of normal tissue [22,23,24], and is associated with poor overall survival (OS) and disease-free survival [22,24], indicating a significant prognostic value for HCC (Table 1). Knockdown of LOX in HCC cells has been reported to suppress proliferation, migration, and invasion, and reduce vascular endothelial growth factor (VEGF) through p38 mitogen-activated protein kinase (MAPK) signaling [22]. Meanwhile, overexpression of LOX in tumor initiating cells (TICs)-enriched HCC enhances tube formation of endothelial cells through secreted VEGF, wherein the stimulated angiogenesis can be blocked by LOX inhibitor β-aminopropionitrile [23]. LOX has been shown to mediate hypoxia-induced cancer metastasis [25]. The LOX expression in HCC cells is upregulated under hypoxia in a hypoxia inducible factor (HIF-1α)-dependent manner [26]. Specifically, hypoxia response elements (HREs) in the LOX gene promoter have been identified [27,28]. In addition, transactivator protein X (HBx), a viral oncoprotein encoded by hepatitis B virus (HBV), activates the HIF-1α/LOX signaling pathway to enhance cross-link collagen in the extracellular matrix (ECM), leading to HCC growth and metastasis [29]. Huang et al. demonstrated that the food components pterostilbene and curcumin suppress migration and invasion induced by long-term ethanol exposure through inhibiting LOX [30]. These results collectively indicate an oncogenic role of LOX in HCC, while demonstrating an anti-tumor effect of LOX-PP. Furthermore, Zheng et al. reported that HCC tissues express a decreased level of LOX-PP as compared to that of normal tissue [31]. Adenovirus-delivered overexpression of LOX-PP in HCC cells enhances apoptosis and represses proliferation, migration, and invasion via the mitogen-activated protein kinase (MAPK) pathway [31].

Taken together, these studies indicate that the upregulation of the LOX level is a predictive sign for HCC. It must be noted however, that the precise role of LOX in CCA remains unclear and thus requires further study.

### 2.2. LOX and Angiogenesis

ECM remodeling is a key aspect involved in the angiogenesis surrounding tumors. A stiff ECM supports angiogenesis with abnormal vasculature, leading to a corrupt TME which promotes tumor dissemination [32]. As an ECM remodeling enzyme, the relevance of LOX in promoting angiogenesis to reshape the TME of HCC has recently been identified by Yang et al. They demonstrated that TICs express higher levels of LOX than the corresponding control cells, and that the LOX expression level correlates positively with VEGFA and VEGFC. LOX secreted by TICs-enriched HCC promotes the tube formation of endothelial cells through the upregulation of VEGF [23]. Overexpression of LOX enhances the extent of angiogenesis, whereas LOX inhibitor β-aminopropionitrile (BAPN) reverses the effect [23]. In addition, LOX inhibitor potentiates the anti-angiogenesis effect of the clinical drug Sorafenib. Zhu et al. also observed a positive correlation between LOX and VEGF in HCC cells, demonstrating that the knockdown of LOX suppresses HCC proliferation, migration, and invasion. With regard to the functional mechanism, LOX acts to mediate TGF-β-induced p38 AMPK-VEGF signaling pathway activity [22]. Interestingly, LOX-PP processed from pro-LOX protein appears to play a tumor-suppressive role on HCC cells. Zheng et al. showed that adenovirus-delivered LOX-PP overexpression in HCC cells hinders cell cycle progression cellular motility, as well as angiogenic activators MMP-2 and MMP-9 [31]. Overall, these reports support the positive role of LOX in angiogenesis. Nevertheless, the exact mechanism underlying LOX-PP perturbation of EC proliferation and vessel formation requires further study. The roles and mechanisms of LOX in liver cancer and its microenvironment are summarized in Table 2.

## 3. Roles of LOXL1 and LOXL3 in Liver Cancer

Several lines of study have highlighted the role of LOXL1 in tumor progression, such as glioma [33], gastric cancer [34], colorectal cancer [35], pancreatic ductal adenocarcinoma (PDAC) [36], yet its effect on liver cancer remains unclear. The biological role of LOXL1 in eye disorders, particularly pseudoexfoliation syndrome and glaucoma, has been recently reviewed in detail by Greene et al. [37]. Nevertheless, the functioning of LOXL1 as a factor in regulating the pathophysiology of the liver is still emerging. Zhao et al. identified an approximately 30-fold increase in the LOXL1 level in a rodent liver fibrosis model [7]. The knockdown of LOXL1 led to mitigation of liver fibrosis in vivo and decreases in fibrogenic markers in the hepatic stellate cell (HSC) line LX-2 in vitro [7]. Meanwhile, Ma et al. found that the knockdown of LOXL1 suppresses proliferation and fibrogenesis induced by transforming growth factor-β1-mothers against the decapentaplegic homolog 2/3 (TGF-β1- Smad2/3) signaling pathway in an HSC line.

Similarly, while the role of LOXL3 in gastric cancer [38], colorectal cancer [39], PDAC [40], and ovarian cancer [41] has been reported, its significance in liver cancer awaits further investigation.

## 4. Role of LOXL2 in Liver Cancer

### 4.1. Prognostic Value and Biological Role of LOXL2 in HCC

The viability of LOXL2 as an excellent diagnostic marker and a potential therapeutic target for HCC has previously been identified by Wong et al. HCC tissue presents higher expression levels of mRNA and immunoactivity of LOXL2 than that of non-tumor tissue. Both early and late stage HCC patients show higher serum LOXL2 levels than non-HCC control subjects. Importantly, serum LOXL2 level has an excellent capacity to distinguish HCC from non-HCC patients, as evidenced by the area under the receiver operating characteristic (ROC) standing at 0.896 [42]. Wang et al. reported an elevated expression of LOXL2 in HCC tissue, significantly associated with poor OS [43]. Furthermore, an RNA interference study in HCC cell lines verified that LOXL2 acts to promote migration, invasion, and EMT, and that LOXL2 is positively regulated by HIF-1α [43]. In addition, Choi et al. reported an elevated LOXL2 expression level in HCC tissue compared to non-tumor tissue, suggesting that LOXL2 positivity may serve as a predictive factor for poor OS and disease-specific survival (DSS) [44]. Meanwhile, Shao et al. reported that high LOXL2 cytoplasmic expression along with VM positivity indicate poor OS and DFC in HCC patients [45]. Overexpression of LOXL2 promotes the EMT transcription factor SNAIL, migration, invasion, and tube formation in HCC cells [45]. Ninomiya et al. noted that the high expression level of LOXL2 in HCC tissue and cell lines exhibited predictive significance for poor DFS and OS [46]. The knockdown of LOXL2 by siRNA has been shown to attenuate proliferation and cell colony formation, and to promote cycle arrest and apoptosis in HCC cells [47]. Mechanistically, it has been reported that HIF-1α and the TGF-β-SMAD4 axis account for the activation of LOXL2 expression in HCC [42,43,48]. These reported investigations reveal that an increase in the LOXL2 level is a representative biomarker for HCC.

### 4.2. LOXL2 in HCC Microenvironment

The central role of LOXL2 in bolstering the formation of TME has been reported in several lines of study (Table 3). Tse and colleagues revealed that the secreted form of LOXL2 driven by the HBx-HIF-1α axis reshapes crosslinking of collagen fibers to facilitate HCC invasion, as evidence by ultrastructural imaging [29]. In addition to LOXL2′s effect on non-cellular components, recent studies have demonstrated its role in boosting angiogenesis through intercellular crosstalk. As such, Fan et al. demonstrated LOXL2 activates the SNAIL/fructose-1,6-biphosphatase (FBP1) axis to upregulate the HIF-1α/VEGF pathway in HCC cells, leading to enhanced tubular networks of endothelial cells [49]. Xing et al. reported that increased matrix stiffness can induce M2 polarization of the THP-1 cell line and increase LOXL2 expression through activation of integrin β5- focal adhesion kinase (FAK), mitogen-activated protein kinase kinase (MEK), 1/2-extracellular signal-regulated kinase (ERK) 1/2 pathway and upregulation of HIF-1α [50].

The formation of a tumor pre-metastatic niche in the distant organ, by which a fertile “soil” assists the settlement of circulating tumor cells, serves as a critical molecular event facilitating the subsequent implementation of distant metastasis [51]. In this regard, the role of LOXL2 in promoting the formation of local and distal metastatic niches has recently been noted. Wong et al. observed that HCC-derived LOXL2 promotes intrahepatic metastasis by increasing tissue stiffness to facilitate HCC cell motility and foster extrahepatic metastasis by enhancing recruitment of bone-marrow-derived cells to the pre-metastatic site in the lung [42]. The study further demonstrated a convergent control of HIF-1α, TGF-β/SMAD4 axis, microRNA-26a (miR-26a), and miR-29a upstream LOXL2 [42]. A study conducted by Wu et al. also supports the role of LOXL2 in assisting the formation of a pro-metastatic niche by facilitating BMDC motility [52]. Increased matrix stiffness upregulates LOXL2 expression by the integrin/JNK/c-JUN signaling pathway in HCC. The HCC-derived LOXL2 upregulates matrix metallopeptidase 9 (MMP-9) expression and fibronectin production in lung fibroblasts and functions as a chemoattractant to increase the invasion of BMDC and HCC [52].

### 4.3. LOXL2 in CCA

The role of LOXL2 in CCA tumor promotion has been highlighted in recent years. In 2014, Xu et al. demonstrated that LOXL2 positivity is associated with CCA lymph node metastasis, differentiation, and poor OS [53]. Knockdown of LOXL2 in CCA cells reduced invasive activity in vitro and liver metastasis in vivo. Furthermore, the expression of LOXL2 was respectively suppressed/augmented by knockdown/overexpression of the 67 laminin receptor (67LR) [53]. Bergeat et al. also found that both mRNA and protein expression of LOXL2 are increased in CCA tumor stromal, and that high strong staining of LOXL2 predicts poor OS and DFS in patients with CCA [54]. Meanwhile, Peng et al. reported that elevated expression of LOXL2 along with interacting factor GATA6 are associated with poor OS and DFS, while positively correlated with VEGFA and microvessel density in human CCA tissue [55]. GATA6 interacting with LOXL2 at the SRCR domain is required for VEGFA transcriptional activity and protein expression of the CCA cell line. The collected conditioned medium verified the positive role of LOXL2/GATA6 in the promotion of endothelial cell tube formation. Knockdown of LOXL2 in CCA cells blunted tumor growth and microvessel density in a xenograft nude mouse model [55]. In addition, silencing of LOXL2 can suppress cell invasion and EMT activity induced by the hepatitis C virus core protein (HCVc) in CCA [56]. Table 3 outlines the mechanisms of LOXL2 in liver cancer and shaping of the hepatocarcinogenic microenvironment.

## 5. Roles of LOXL4 in Tumor Microenvironment and Progression

Recently, research has shed light on the prediction value and supportive function of LOXL4 on shaping the hepatocarcinogenic microenvironment. Through multiple datasets, Li el at. revealed that the mRNA level and immunoreactivity of LOXL4 upregulates in HCC tissue, and that high LOXL4 protein expression predicts poor OS, DFS, and cumulative survival rate, and serves as an independent predictor for tumor size and TNM stage [57]. The in vitro study indicated that TGF-β, but not hypoxia, can induce LOXL4 upregulation in HCC cell lines, including SMMC-7721, SK-Hep1, Huh7, and Hep3B. Further study employing gain- and loss-of-function, both in vitro and in vivo, confirmed the pro-metastatic role of LOXL4 in HCC progression [57]. On one hand, the exosome-mediated secretion of HCC cells, rich in LOXL4, to adjacent HCC, activates the focal adhesion kinase/steroid receptor coactivator (FAK/Src) pathway through a hydrogen peroxide (H_2_O_2_)-mediated mechanism, leading to enhanced HCC migration ability. On the other hand, intercellular transfer of exosome-LOXL4 from HCC to human umbilical vein endothelial cells (HUVEC) promotes angiogenesis, as evidenced by increased HUVEC migration and tube formation [57]. More recently, Tan et al. identified LOXL4 as a key factor in forming an immunosuppressive microenvironment for HCC [15]. The expression of LOXL4 was increased in a mouse model with liver carcinogenesis induced by a choline-deficient, L-amino acid-defined (CDAA) diet. LOXL4 acts to promote macrophage infiltration into the liver to support tumor growth. Specifically, LOXL4-harboring exosomes are primarily internalized in hepatic macrophages to shape an immunosuppressive phenotype, such as up-regulated programmed cell death 1 ligand 1 (PD-L1) expression, which further acts to suppress the activity of CD8^+^ T cells. The mechanism underlying the immunosuppressive role of LOXL4 on shaping macrophage phenotype lies in an H_2_O_2_-dependent activation of the interferon alpha and beta receptor subunit-1-signal transducer and activator of transcription 1 and 3-programmed death-ligand 1 (IFNAR1-STAT1/3-PD-L1) pathway [15]. The emerging role of LOXL4 in guiding the progression and modeling the microenvironment of HCC is summarized in Table 4.

It must be noted that there are studies indicating a contradictory point of view regarding the role of LOXL4 in HCC. Shao et al. demonstrated that low LOXL4 expression in patients’ liver cancer tissue predicts poor OS [58]. LOXL4 expression was found to display a positive correlation with tumor suppressor p53. More specifically, the employment of a genome-wide clustered, regularly interspaced, short palindromic repeats (CRISPR) screen identified the anti-HCC effect of 5-azacytidine (5-aza-CR), depending on LOXL4-mediated reactivation of p53, which relays apoptosis in HCC cells. The mechanism accounting for the 5-azacytidine-induced LOXL4-p53 axis of HCC cells lies in the binding between D677/D679 in LOXL4 and K381/382 in p53, noted to occur in other cancer types, including lung, breast, and ovarian cancers, as well as melanoma [58]. Furthermore, Tian et al. found that both mRNA and protein expression levels of LOXL4 were lower in HCC tissue than that of peritumoral tissue [59]. The lower LOXL4 expression is associated with poor OS and higher cumulative recurrence rates. HCC cell lines with high metastatic potential exhibit a lower level of LOXL4 mRNA expression than those with low metastatic potential [59]. In light of the aforementioned studies, further investigation is indeed required to more precisely clarify the functions and mechanisms of LOXL4. In addition, further study to determine the potential role of LOXL4 in CCA is warranted.

## 6. Regulatory Pathways at Various Sub-Cellular Levels and the Impact of Genetic/Epigenetic Perturbations

Given that the expression and intercellular crosstalk of LOX, LOXL2, and LOXL4 play critical roles in TME formation and the progression of HCC, elucidation of their regulatory pathways may identify promising therapeutic targets. With regard to modulation at the transcriptional level, HIF-1 acts as a critical transcription factor directly targeting the *LOX*, *LOXL2*, and *LOXL4* genes to upregulate their expression [13,60,61]. Umezaki et al. demonstrated that siRNA-based knockdown of HIF-1 considerably reduces LOX expression HCC cells [24]. The HIF-LOX axis acts to dictate the expression of EMT activators TWIST, VIMENTIN, and SLUG [24]. The HIF-1α/LOX axis also mediates HBx-induced HCC progression [29]. HIF-1α upregulates LOXL2 to promote HCC cell proliferation, migration, invasion, and the EMT markers E-cadherin and Vimentin [43]. Moreover, Wong et al. showed that the TME-fostering effect of LOXL2 relies on the activation of the HIF-1α/TGF-B/SMAD4 pathway [42]. Interestingly, LOXL2 and HIF-1α are thought to exert reciprocal modulation; as such, Fan et al. showed that LOXL2 can act to upregulate HIF-1α through the Snail-FBP1 axis [49]. In addition, the forkhead box M1b (FoxM1b), a transcription factor which exerts a pro-tumor effect on HCC, has been reported to play a positive role in LOX and LOXL2 expression. Park et al. demonstrated that FoxM1b exerts transcriptional activation by directly binding to the promoter of LOX and LOXL2. This effect activates downstream in the AKT-SNAIL pathway which activates the EMT program to drive liver fibrosis and HCC metastasis [62].

At the post-translational level, GATA6 interacts with LOXL2 in promoting angiogenesis and CCA tumor growth. Peng et al. demonstrated that GATA6 binds to LOXL2 at its SRCR domain to subsequently upregulate VEGFA expression and secretion [55]. Of note, matrix stiffness is an external factor that upregulates LOXL2 expression. Wu et al. reported that the integrin β1/α5/JNK/c-JUN axis dictates the expression of LOXL2. The secreted form for LOXL2 further activates AKT to boost the secretion of fibronectin/MMP9/CXCL12 to assist in the formation of a pre-metastatic niche for HCC progression [52].

**Figure 3:**
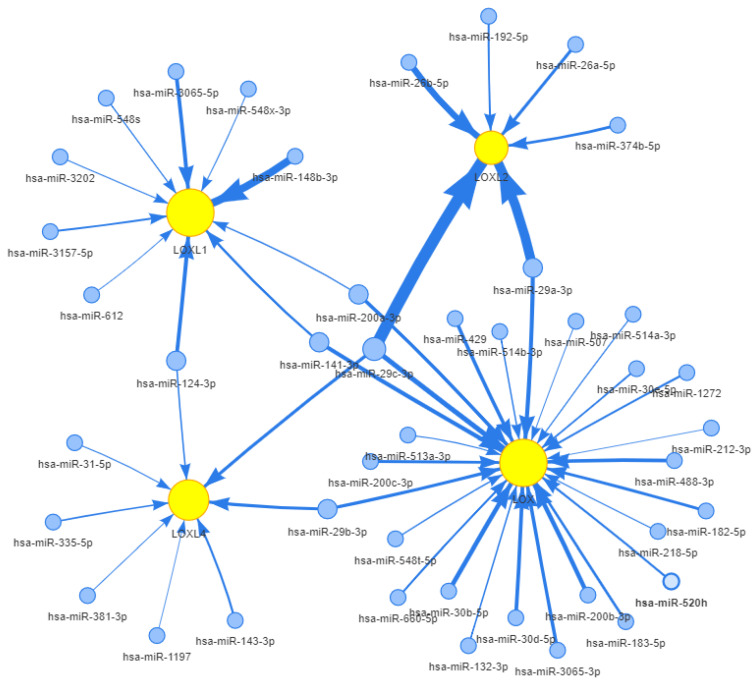
miRs regulatory network of LOX, LOXL1, LOXL2, and LOXL4.

MiRs function as central regulators of gene expression at the post-transcriptional level. In this study, we employed the GSCALite web server to present the miR regulatory network of LOX, LOXL1, LOXL2, and LOXL4 (Figure 3); notably, miR-26a and miR-29a have been identified as negative regulators of LOXL2 in HCC [42]. In addition, the roles of other miRs in suppressing LOX family members in various types of cancer have been revealed, including in neck squamous cell carcinoma [63], prostate cancer [64], renal cell carcinoma [65], breast cancer [66], anaplastic thyroid cancer [67], lung squamous cell carcinoma [68], non-small cell lung cancer [69], and giant-cell carcinoma of the lung [70] (Table 5).

In terms of the impact of genetic alteration, an Arg158Gln substitution in LOX-PP has been shown to be associated with susceptibility of breast [71], lung [72], colorectal [73], and ovarian cancer [74]. Cueva et al. showed that in knock-in strain LOX-PP^Gln^ mice that harbor an Arg152Gln substitution, corresponding to the human Arg158Gln polymorphism of LOX-PP, manifest increased susceptibility to carcinogen-induced breast cancer and hepatic inflammation compared to their wild type counterparts [75]. With reference to the epigenetic episode, Shao et al. demonstrated that 5-azacytidine (5-aza-CR), acting on hypomethylation of DNA by inhibiting DNA methyltransferase, induces LOXL4 upregulation and triggers LOXL4-depedent cell apoptosis in HCC [58].

## 7. Therapeutic Potential of Targeting Approaches on LOX Family Members

To date, a couple of drugs targeting LOX family members are in the early stage of clinical trials, including those focused on pancreatic and colorectal adenocarcinoma [76,77]. However, most clinical trials engaged in LOX family member-targeting drugs for liver cancer are still lacking, based on information issued by ClinicalTrials.gov (https://clinicaltrials.gov/ct2/home). To further explore the potential applications of approaches targeting the LOX family members, we conducted a survey of drugs and approaches reported in preclinical and clinical settings (Table 5).

β-aminopropionitrile (BAPN), an irreversible inhibitor of catalytic activity of LOX and LOX1-4 [78,79,80,81,82], has been shown to exert a suppressive effect on metastatic colonization of circulating breast cancer cells [83], hypoxia-induced invasion of cervical cancer cells [84], and the angiogenic capacity of HUVEC [85]. Despite these reports underscoring BAPN’s role in combating tumors, its dual action on tumor promotion and suppression in a context-dependent manner in prostate cancer [86] should be taken into consideration. BAPN has been reported to hamper the TME that constitutes cross-talk between cancer-associated fibroblast-gastric cancer, leading to attenuated liver metastasis [87]. With regard to HCC, BAPN has been shown to block HCC-promoted proliferation and tube formation of endothelial cells in vitro and suppress angiogenesis and tumor growth in vivo [23]. Ninoyama et al. showed that BAPN inhibits LOXL2 to impede the ability of migration and invasion of HCC cells [46]. In addition, Liu et al. demonstrated the ameliorative effect of BAPN on liver fibrosis induced by CCl_4_ [88]. GW4869, a N-SMase inhibitor that blocks exosome generation [89,90,91,92], has been used to block intercellular exosome-LOXL4 transfer and reduce the cell migratory ability of HCC cells [57]. DNA demethylation small molecule 5-aza-CR exhibits a suppressive effect on tumor growth and cell proliferation by triggering the LOXL4-p53 signaling pathway to activate the expression of pro-apoptotic genes, p53 inducible gene 3 (*PIG3*) and Bcl-2-associated X protein (*BAX*) [58]. Thus, in addition to synthetic compounds, LOX-inhibiting phytochemicals may be potential candidates for the treatment of liver cancer. To illustrate, Huang et al. reported that pterostilbene/curcumin analogues exert a LOX-inhibiting effect to attenuate the migration and invasion of HCC [30].

The LOXL2-neutrolazing monoclonal antibody AB0023 exerts the inhibitory effect of LOXL2 by targeting its SRCR domain [93]; whereby, AB0023 has been shown to effectively alleviate liver fibrosis in mouse models induced by CCl_4_ [94], by thioacetamide (TAA), and using Mdr2^−^/^−^ plus 3,5-diethoxycarbonyl-1,4-dihydrocollidine (DDC) [8]. Interestingly, Simtuzumab (AB0024/GS-6624) is the humanized version of AB0023, which has been tested in phase II clinical trials directed at colorectal adenocarcinoma, pancreatic adenocarcinoma, and primary sclerosing cholangitis [76,77,95]. Furthermore, PXS-5153A, a dual LOXL2/LOXL3 inhibitor developed by Schilter et al., has been shown to ameliorate liver fibrosis in a CCl_4_ model and in a streptozotocin plus high fat diet-induced steatohepatitis model [96]. The first selective inhibitor for LOXL2, LOXL2-IN-1 hydrochloride [97], has recently been identified to act to suppress Snail, HIF-1α, and VEGF, which are promotion factors in HCC invasion and angiogenesis [49].

A number of LOX-targeting drugs which have recently been reported to feature anti-cancer efficacy are likely to serve as candidates in the treatment of liver cancer. Springer et al. demonstrated that the LOX inhibitor CCT365623, bearing an aminomethylenethiophene (AMT) scaffold, has exhibited anti-metastasis efficacy in a LOX-driven spontaneous breast cancer model [98]. The team soon after suggested a series of 2-aminomethylene-5-sulfonylthiazole (AMTz) as dual inhibitors of LOX and LOXL2 [99]. One of the AMTz-bearing inhibitors, AMTz-21b, effectively suppressed tumor growth in their spontaneous breast cancer mouse model [99]. Meanwhile, dextran sulfate (DS) acts to down-regulate the expression of LOX to suppress invasive and migratory behaviors in gastric cancer cells [100]. The combination of DS and BAPN exerts a superior effect compared to DS or BAPN alone [100]. In addition, salidroside, a phenylpropanoid glycoside isolated from *Rhodiola rosea* L, down-regulates the mRNA expression level of LOX, LOXL1-4, and HIF-1α in a dose-dependent manner in pancreatic cancer cells [101]; thereby, the invasive activity of cancer cells, xenograft tumor growth, and distal metastasis can be hindered by treatment with salidroside [101]. Further studies have indicated that Escin Ia, belonging to a subclass of the saponin fraction of *Aesculus chinensis* Bunge fruits (SFAC), acts towards the down-regulation of LOXL2 and the inhibition of the metastatic behavior of triple-negative breast cancer in vitro and in vivo [102]. Escin Ia perturbs the EMT program, as evidenced by enhanced E-cadherin and curbed Vimentin and alpha smooth muscle actin (α-SMA), as well as transcription factors Snail, Slug, Zeb1, Zeb2, and Twist [102]. Meanwhile, ammonium tetrathiomolybdate (TM), acting as copper chelator, has been shown to suppress LOX activity, cell proliferation, and the bone destruction behavior of head and neck squamous cell carcinoma (HNSCC) [103]. Finally, Hutchinson et al. indicated that (2chloropyridin-4-yl) methanamine 20 is the most potent compound for LOXL2, amid two series of novel LOXL2 enzyme inhibitors, benzylamines substituted with electron withdrawing groups at the *para*-position and 2-substituted pyridine-4-ylmethanamines [97].

MiRs are approximately 22 nucleotides in length, short non-coding RNAs, functioning in a pathway-centric manner by targeting multiple genes, and are potential therapeutic targets for liver cancer [104]. In recent years, there has been growing evidence elucidating the pathway-centric manner of microRNAs (miRs) on the modulation of the LOX family members in carcinogenesis. More specifically, Wong et al. found that miR-29a-3p and miR-26a-5p bind to the 3′untranslated region (3′UTR) of LOXL2 mRNA, leading to suppression of LOXL2 expression, which is essential for the promotion of TME and the formation of a pre-metastatic niche in HCC [42]. In addition, Seki’s team have demonstrated that a set of miRs, miR-26a/b, miR-29a/b/c, and miR-218, significantly inhibit metastasis by down-regulating LOXL2 mRNA in HNSCC [63]; demonstrating similar findings in prostate cancer [64]. In renal cell carcinoma (RCC), miR-26a/b overexpression exhibits inhibitory efficacy on cancer cell proliferation, migration, and invasion through the direct binding of 3′UTR of LOXL2 mRNA [65]. Saatci et al. identified that miR-142-3p exerts an inhibitory role on LOX expression for overcoming chemoresistance in triple-negative breast cancer [66]. In anaplastic thyroid cancer (ATC), Boufraqech et al. reported that miR-30a interacts with the 3′UTR of LOX to mediate anti-tumor efficacy, as evidenced by suppressing cell invasion and migration, EMT markers expression, LOX expression, and metastatic capacity [67]. In lung squamous cell carcinoma (LSCC), miR-29a/b/c restricts cell migration and invasion by binding to the 3′UTR of LOXL2 and prevents its transcription [68]. Kamikawaji et al. identified that miR-29a exerts an anti-aggressive effect on lung cancer cells and an anti-proliferation effect in lung fibroblasts by directly binding to LOXL2 [69]. In non-small cell lung cancer (NSCLC), miR-504 has been reported to function as a tumor-suppressing factor by directly targeting the 3′UTR of LOXL2 [105]. Furthermore, Duan et al. found that miR-30b reduces LOX expression by directly interacting with the 3′UTR of LOX in lung cancer cells [70]. Several lines of study have revealed the tumor-promoting role of particular miRs. To illustrate, miR-135a-5p presents a tumor-promoting role as evidenced by in vitro and in vivo studies directly targeting LOXL4 [106]. Additionally, miR-210 has been found to promote lung cancer cell proliferation, colony formation, migration, and invasion via targeting LOXL4 [107].

## 8. Future Perspective

**Figure 4:**
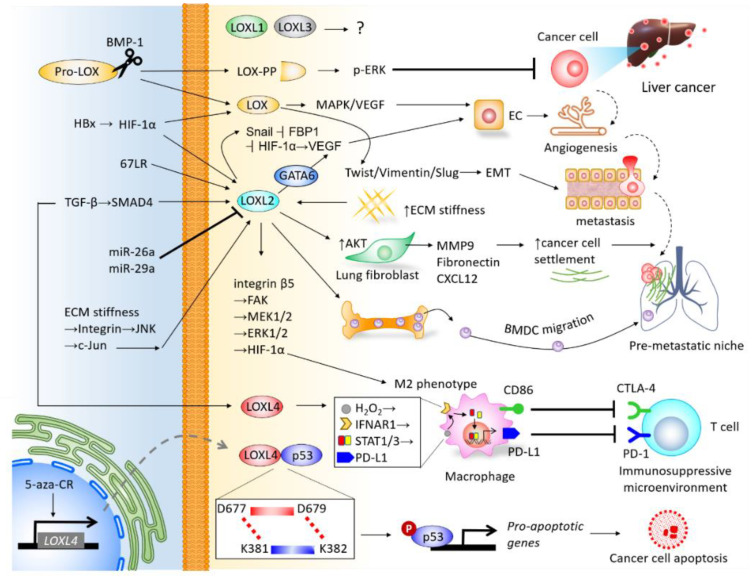
Integrative network depicting the biological roles of LOX family members in the tumor microenvironment (TME) of liver cancer.

The LOX family members are responsible for remodeling the cross-linking of structural ECM. There is mounting clinical evidence indicating their significance in predicting prognosis and diagnosis, and their roles in promoting cancer cell proliferation, invasiveness, and shaping the TME of liver cancer (Figure 4), particularly LOX, LOXL2, and LOXL4. As the majority of current studies focus on HCC, insight into the mechanisms underlying LOX family members in CCA requires further investigation. Furthermore, the roles of LOXL1 and LOXL3 in the pathogenesis of liver cancer remain unclear, which also necessitates further study. It is important to note that drugs developed to target LOX family members have been effective at inhibiting the progression of HCC in preclinical models, and have shown efficacy in clinical trials of other cancer types. Investigations into miRs-dictated mechanisms for the activity of LOX family members could further shed light on the molecular activity of TME and pave the way to prospective clinical therapeutic approaches. To summarize, LOX family members represent attractive therapeutic targets for the treatment of liver cancer.

## Figures and Tables

**Table 1:** Clinical relevance of LOX family members in liver cancer.

LOX Family Member	Patients	Expression Level in Tumor	Clinical Relevance	PMID
LOX	HCC	Up	Prognostic marker for high recurrence rate and poor OS	30919528
	HCC	Up	Prognostic marker for poor OS and DFS	26048020
LOXL2	HCC	Up	Prognostic marker for poor OS	28449718
	HCC	Up	Prognostic marker for poor OS and DFS	29620290
	HCC	Up	Prognostic marker for poor OS and RFS	29938458
	HCC	Up	Prognostic marker for poor OS and DFS	30506621
	CCA	Up	Prognostic marker for poor OS and DFS	31322171
	CCA	Up	Prognostic marker for poor OS and DFS	27363654
	HCC	Up	Serum LOXL2 as an excellent differential marker	25048396
LOXL4	HCC	Up	Prognostic marker for poor OS	33068461
	HCC	Up	High LOXL4 indicates poor OS and DFS	30704479
	HCC	Down	High LOXL4 indicates high recurrence rate and poor OS	26097573

CCA, cholangiocarcinoma; DFS, disease-free survival; HCC, hepatocellular carcinoma; OS, overall survival; RFS, recurrence-free survival.

**Table 2:** Roles and mechanisms of LOX in hepatocarcinogenic progression and the microenvironment.

Liver Cancer Context	Donor or Approach	Recipient	Signaling Pathway	Biological Activity	PMID
HCC cell line (SK-hep-1, MHCC-97-H, Hep-G2, SSMC-7721, Huh7)	--	--	LOX→MAPK→VEGF	↓ proliferation; ↓migration; ↓ invasion	26048020
HCC cell line (SK-hep-1, Hep-G2)	Ad-LOX-PP	HCC	LOX-PP→p-ERK LOX-PP┤MMP-2, MMP9	↑ apoptosis; ↓ proliferation ↓ migration; ↓ invasion	24573150
Xenograft using Sphere-derived TIC (HuH-7)	TIC	EC	LOX→ VEGF	↑ angiogenesis	30720077
HCC cell line (SK-hep-1)	--	--	HIF1→LOX→ TWIST/Vimentin/Slug	↑ EMT ↑ cell migration and invasion	30919528

“↓”: suppress; ”↑”: promote; →”: activate; “**┤**”: inhibit; Ad-LOX-PP, adenovirus-delivered lysyl oxidase propeptide; EC, endothelial cell; EMT, epithelial–mesenchymal transition; p-ERK, phosphorylated extracellular signal-regulated kinase; HIF, hypoxia inducible factor; LOX, lysyl oxidase LOX-PP, lysyl oxidase propeptide; TIC, tumor initiating cells; VEGF, vascular endothelial growth factor.

**Table 3:** Roles and mechanisms of LOXL2 in the hepatocarcinogenic microenvironment.

Liver Cancer Context	Donor or Stimulant	Recipient	Signaling Pathway	Biological Activity	PMID
HCC cell lines (Huh7 and Hep3B)	HCC	EC	LOXL2→ Snail ┤FBP1┤HIF-1α→VEGF	↑ Proliferation; ↑ glycolysis; ↑ angiogenesis	32323822
Mφ (THP-1 cell ine)	↑ Matrix stiffness	Mφ	integrin β5 →FAK→MEK1/2-ERK1/2→ HIF-1α→LOXL2	↑ M2 phenotype of Mφ	32964626
HBx-transfected HCC (HepG2, Hep3B, MHCC97L)	HCC	collagen	HBx→HIF-1α→LOXL2	↑ ECM collagen cross-link ↑ Cell invasion ↑ Tumor growth ↑ Metastasis	29799025
HCC cell line (HepG2)	--	--	HIF-1α→LOXL2→E-cadherin/Vimentin	↑ EMT ↑ cell migration and invasion ↑ Vasculogenic mimicry	28449718
HCC cell lines (MHCC97L)	HCC	Collagen; BMDC	HIF-1α→LOXL2 TGF-β→SMAD4→LOXL2 miR-26a ┤LOXL2 miR-29a ┤LOXL2	↑ tissue stiffness ↑ local metastasis ↑ BMDC requitement to pre-metastatic site ↑ distant metastasis	25048396
HCC cell lines (MHCC97L)	HCC	Lung fibroblast; BMDC	↑ Matrix stiffness→ integrin→JNK→c-Jun→LOXL2 (HCC) LOXL2→AKT→MMP9, CXCL12 (lung fibroblast)	↑ HCC adhere to lung fibrobalst ↑ BMDC migration ↑ HCC migration	29728125
CCA cell lines (QBC939 and RBE)	CCA	EC	LOXL2-GATA6 interaction→ VEGFA	↑ Tumor growth ↑ angiogenesis	31322171
QBC939	CCA	--	67LR→LOXL2	↑ cell invasion ↑ tumor metastasis	24794791

”↑”: promote; “→”: activate; “**┤**”: inhibit; 67LR, 67 laminin receptor; BMDC, bone marrow-derived cells; EC, endothelial cell; ERK, extracellular signal-regulated kinase; FAK, focal adhesion kinase; HIF, hypoxia inducible factor; HUVEC, human umbilical vein endothelial cells; MEK, mitogen-activated protein kinase kinase; Mφ, macrophage; SMAD, mothers against decapentaplegic homolog; Src, steroid receptor coactivator; TGF-β, transforming growth factor beta; VEGFA, vascular endothelial growth factor A.

**Table 4:** Roles and mechanisms of LOXL4 in hepatocarcinogenic progression and the microenvironment.

Liver Cancer Context	Donor	Recipient	Signaling Pathway	Biological Activity	PMID
Mouse treated with CDAA diet and CCl_4_	HCC	Mφ	LOXL4 (HCC)→H_2_O_2_→IFNAR1→STAT1/3→PD-L1 (Mφ) ┤ cytotoxicity (T cell)	↑ Immunosuppressive phenotype of Mφ ↓ T cell cytotoxicity; ↑ Tumor growth	33068461
HCC cell line xenograft	HCC	HCC	TFG-β→exosomal LOXL4→FAK→Src	↑ cell migration and invasion ↑ intrahepatic and pulmonary metastasis	30704479
	HCC	EC	Exosomal LOXL4→ HUVEC migration and tube formation	↑ Angiogenesis	30704479

“↓”: suppress; ”↑”: promote; “→”: activate; “**┤**”: inhibit; CCl_4_, carbon tetrachloride; CDAA, choline-deficient L-amino acid-defined; EC, endothelial cell; FAK, focal adhesion kinase; HUVEC, human umbilical vein endothelial cells; IFNR1, interferon alpha and beta receptor subunit-1; Mφ, macrophage; PD-L1, programmed death-ligand 1; Src, steroid receptor coactivator; STAT, signal transducer and activator of transcription; interferon alpha and beta receptor subunit-1;TGF-β, transforming growth factor beta.

**Table 5:** Inhibitors targeting LOX family members in preclinical models.

Agents	Biological Property	Targets of Action	Disease Model	PMID
BAPN	Small-molecule inhibitor	(-) LOX, LOXL1-4	HCC	30720077
		(-) LOXL2	HCC	29620290
		(-) LOX	Liver metastasis of GC	31678002
		(-) LOX, LOXL1-4	Liver fibrosis	26700732
GW4869	N-SMase inhibitor	Exosome-mediated transfer of LOXL4	HCC	30704479
pterostilbene/curcumin analogues	Stilbene/curcuminoids compounds	(-) LOX	HCC	23560895
AB0023	mAb	(-) LOXL2	Liver fibrosis	28073888
		(-) LOXL2	Liver fibrosis	20818376
LOXL2-IN-1 hydrochloride	Small-molecule inhibitor	(-) LOXL2	HCC	32323822
PXS-5153A	Small-molecule inhibitor	(-) LOXL2/3	Liver fibrosis	30536539
5-aza-CR	DNA methylation Inhibitor	(+) LOXL4	HCC	30728460
CCT365623	Small-molecule inhibitor	(-) LOX	Lung metastasis of BC	31070916
AMTz-21b	Small-molecule inhibitor	(-) LOX, LOXL2	Lung metastasis of BC	31430136
Salidroside	Glucoside of tyrosol	(-) LOX, LOXL1-4	Lung metastasis of PC	31162697
escin Ia	Subclass of SFAC	(-) LOXL2	Lung metastasis of BC	27008697
ammonium tetrathiomolybdate	Copper chelator	(-) LOX	Bone invasion of HNSCC	29328370
miR-26a, miR-29a	Non-coding RNAs	(-) LOXL2	HCC	25048396
miR-26a/b, miR-29a/b/c, miR-218	Non-coding RNAs	(-) LOXL2	HNSCC	26490187
miR-26a/b, miR-29a/b/c, miR-218	Non-coding RNAs	(-) LOXL2	PC	27278788
miR-26a/b	Non-coding RNAs	(-) LOXL2	RCC	26983694
miR-142	Non-coding RNAs	(-) LOX	BC	32415208
miR-30a	Non-coding RNAs	(-) LOX	ATC	25488748
miR-29a/b/c	Non-coding RNAs	(-) LOXL2	LSCC	26676674
miR-29a	Non-coding RNAs	(-) LOXL2	NSCLC	27488440
miR-30b	Non-coding RNAs	(-) LOX	GCCL	31093946
miR-135a	Non-coding RNAs	(-) LOXL4	NSCLC	30993701
miR-504	Non-coding RNAs	(-) LOXL2	NSCLC	29156517

(-) inhibit; (+) activate; 5-aza-CR, 5-azacytidine; ATC, anaplastic thyroid cancer; BAPN, beta-aminopropionitrile; BC, breast cancer; GC, gastric cancer; GCCL, giant-cell carcinoma of the lung; HNSCC, head and neck squamous cell carcinoma; LSCC, lung squamous cell carcinoma; NSCLC, non-small cell lung cancer; PC, prostate cancer; RCC, renal cell carcinoma; SFAC, saponin fraction of *Aesculus chinensis* Bunge fruits.
